# Forces acting on codon bias in malaria parasites

**DOI:** 10.1038/s41598-018-34404-9

**Published:** 2018-10-29

**Authors:** I. Sinha, C. J. Woodrow

**Affiliations:** 1 Mahidol-Oxford Tropical Medicine Research Unit (MORU), Mahidol University, Bangkok, Thailand; 2 Centre for Tropical Medicine and Global Health, University of Oxford, Oxford, UK

## Abstract

Malaria parasite genomes have a range of codon biases, with *Plasmodium falciparum* one of the most AT-biased genomes known. We examined the make up of synonymous coding sites and stop codons in the core genomes of representative malaria parasites, showing first that local DNA context influences codon bias similarly across *P*. *falciparum*, *P*. *vivax* and *P*. *berghei*, with suppression of CpG dinucleotides and enhancement of CpC dinucleotides, both within and aross codons. Intense asexual phase gene expression in *P*. *falciparum* and *P*. *berghei* is associated with increased A3:G3 bias but reduced T3:C3 bias at 2-fold sites, consistent with adaptation of codons to tRNA pools and avoidance of wobble tRNA interactions that potentially slow down translation. In highly expressed genes, the A3:G3 ratio can exceed 30-fold while the T3:C3 ratio can be less than 1, according to the encoded amino acid and subsequent base. Lysine codons (AAA/G) show distinctive behaviour with substantially reduced A3:G3 bias in highly expressed genes, perhaps because of selection against frameshifting when the AAA codon is followed by another adenine. Intense expression is also associated with a strong bias towards TAA stop codons (found in 94% and 89% of highly expressed *P*. *falciparum* and *P*. *berghei* genes respectively) and a proportional rise in the TAAA stop ‘tetranucleotide’. The presence of these expression-linked effects in the relatively AT-rich malaria parasite species adds weight to the suggestion that AT-richness in the *Plasmodium* genus might be a fitness adaptation. Potential explanations for the relative lack of codon bias in *P*. *vivax* include the distinct features of its lifecycle and its effective population size over evolutionary time.

## Introduction

Codon bias, a generalised tendency to use codons non-randomly, occurs both between and within individual organisms^1^. From the time of its discovery, adaptive explanations have been proposed, with early studies on *E*. *coli* ribosomal protein genes leading to a hypothesis that bacterial codon usage was an adaptive genome strategy for optimal translational efficency and/or accuracy^2–4^. Studies within individual organisms indicate that codon bias can influence transcription^5^, mRNA editing and stability^6^, translation initiation and elongation^7,8^, and post-translational modification^9^. Increased translational speed is often attributed to adaptation of codons to the tRNA pool^7,8,10^ but codon adaptation may also reflect selection for avoidance of ‘wobble’ codon-tRNA interactions (which slow down translation^11^) and frameshift-prone sequences.

In contrast, explanations for underlying biases in base composition across entire genomes (AT- or GC-richness) have tended to revolve around ‘neutral’ mechanisms^12,13^, particularly with smaller effective population sizes in which selection has a relatively smaller role. For example, although a number of functional explanations for the substantial differences in AT-richness among prokarya have been proposed, there is good evidence for a common mutational pressure towards low GC^14^.

Studying codon bias in malaria parasites is of potential importance for several reasons. Malaria is the most important parasitic disease of humans, causing over 200 million cases per year with around half a million deaths^15^. A detailed understanding of malaria biology is considered vital for control and elimination of these parasites, and the complete genome sequences of the key malaria species *Plasmodium falciparum*^16^ and *Plasmodium vivax*^17^ have proved a foundation for a wide range of basic and applied studies relevant to development of new drugs and vaccines^18^. Furthermore, these sequences are now linked to an extensive range of genome-wide studies of transcription and translation using both microarray and next-generation sequencing approaches^19–24^, providing powerful datasets for integrated analysis of the association between expression and codon bias both within and across these important human pathogens. Finally, malaria parasites have distinctive intrinsic codon usage properties. *P*. *falciparum* has one of the most AT-rich genomes known; over the entire genome A or T nucleotides constitute 80.6% of all bases^16^, and 85% of synonymous positions^17^ with non-synonymous positions also affected^25^. The ‘rodent’ malarias (*P*. *berghei*, *chabaudi* and *yoelii*) are evolutionary distant, but nearly as AT-biased (%A + T = 75.6–77.4%) while the (A + T) genome composition of *P*. *vivax* is 62.4%^26^.

Relatively few studies have examined how codon bias varies between genes (or different parts of genes) within individual malaria parasite species. Evidence of expression-associated codon bias has already been described for *P*. *falciparum* and attributed to translational selection favouring particular codon-tRNA interactions^27,28^. Nucleotide context also appears important, with suppression of CpG dinucleotides first reported 30 years ago in *P*. *falciparum*^29–31^ where it is present on a genome-wide scale^32^ and proposed to result from CpG methylation and subsequent deamination of methylcytosine to T, a common property of many eukaryotic organisms^33^.

In order to explore how the various mutational and selective forces underlying codon bias operate within and between malaria parasites, we undertook a genome-wide analysis of synonymous sites and stop codons in three representative malaria parasites (*P*. *falciparum*, *P*. *vivax* and *P*. *berghei*), exploring the relationship between local DNA context and codon bias. Then we explored the influence of the level of gene expression in the asexual phase of the lifecycle. Given the greater expression-related effects observed for *P*. *falciparum* and *P*. *berghei*, we studied how these influences act in combination to influence codon bias in these species. Finally we consider the implications of the association between expression-related bias and AT-richness in the *Plasmodium* genus.

## Methods

### Genomic data

Coding sequences were downloaded from Plasmodb with analysis undertaken in custom Python packages and in R. To aid comparison across species, *P*. *falciparum* 3D7 sequences were chosen that avoided multigene families, producing a set of 5055 coding genes corresponding to the ‘core’ genome, containing a total of approximately 2.6 million synonymous nucleotides i.e. just over 20% of total coding sequence^16^. Coding sequences orthologous to these genes (defined via PlasmodDB) were examined for *P*. *berghei* (4496 genes) and *P*. *vivax* (4712 genes).

### Asexual stage expression data

Data for *P*. *falciparum*, *P*. *berghei* and *P*. *vivax* were obtained from published datasets (Table 1). RNA-Seq data^20,22,23^ were used for the main analyses; microarray data^19,21^ for *P*. *falciparum* and *P*. *vivax* were also checked for consistency. In addition the relationship between *P*. *falciparum* stop codon usage and RNA translation as assessed by ribosome profiling^24^ was studied. For each dataset, genes were ranked by maximum expression level across the lifecycle and then stratified into three levels; I: <75^th^ percentile, II: 75–97^th^ percentile, III: >97^th^ percentile.

**Table 1:** Expression datasets used in the work.

Organism	Type of data	Number of genes with data	Reference
*P*. *falciparum*	Microarray	4555	^19^
*P*. *falciparum*	RNA-seq	4802	^20^
*P*. *falciparum*	Ribosome profiling	3605	^24^
*P*. *vivax*	Microarray	4400	^21^
*P*. *vivax*	RNA-seq	4981 (SMRU1)	^22^
*P*. *berghei*	RNA-seq	4979	^23^

For comparisons of proportions between categories the Chi-squared test was undertaken; p values are shown in Supplementary data.

The parameter for strength of selected codon usage bias, *S* (which is ‘confounded’ by effective population size) was calculated for the nine two-fold synonymous codons (e.g. TTT and TTC coding for phenylalanine) using the formula described by dos Reis and Wernisch^34,35^: *S* = ln(P_hx_/(1-P_hx_)) - ln(P_ref_/(1-P_ref_)), where P_hx_ is the observed frequency of the codon with relatively higher expression in top expression band III, and P_ref_ is its observed frequency in expression band I (i.e. P_hx_ is higher than P_ref_).

## Results

### Context-dependent codon bias

#### Between codon effects

The clearest view of the influence of neighbouring nucleotide context on codon bias can be obtained by examining how the third position in 2-fold or 4-fold synonymous codons varies according to the identity of the first nucleotide of the following codon (termed N_1_)^8^. Because N_1_ lies in the next codon, any patterns observed are unlikely to be due to translational selection (although other selective forces are possible).

#### 2-fold synonymous sites (9 amino acids)

**Figure 1:**
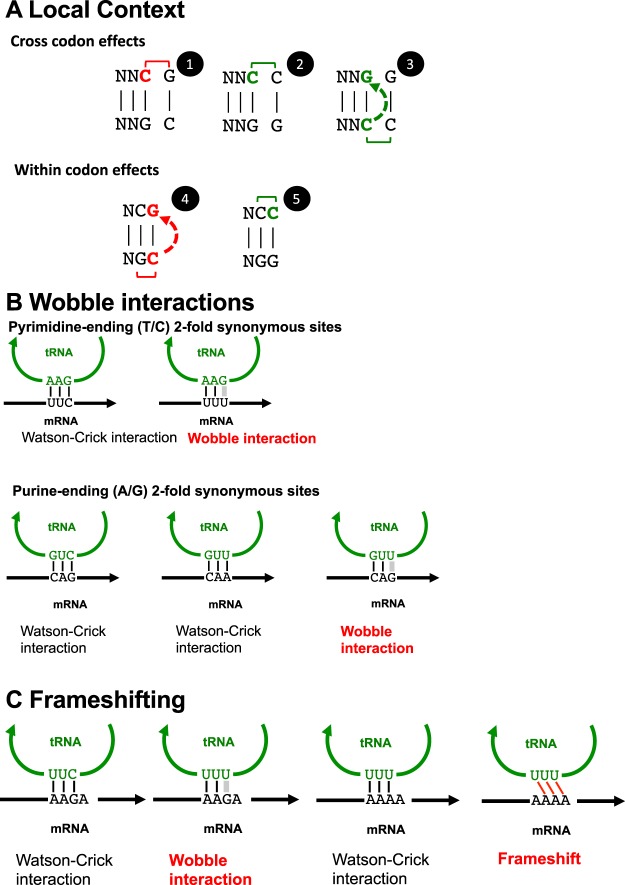
Proposed mechanisms of codon bias described. (**A**) Contextual effects. Across codons (free from translational selection) cytosines on the positive strand followed by an N_1_ guanine (CpG) are suppressed (1) while cytosines followed by an N_1_ cytosine (CpC) are protected (2). Guanines followed by an N_1_ guanine are also more common, possibly reflecting protection of the complementary negative strand cytosine by its 5′ cytosine (3). 4-fold synonymous codons containing C2 (alanine, proline, threonine) show lower proportions of guanine in the 3^rd^ position than glycine and valine, consistent with CpG suppression on the negative strand (4). These C2-containing amino acids also show higher proportions of C3 consistent with protection by the 5′ cytosine (5) on the positive stand. Key: Cytosines within CpG pairs are shown in red while cytosines within CpC pairs are shown in green; dotted arrows indicate effects deriving from the negative strand. (**B**) Avoidance of wobble tRNA-codon interactions in highly expressed genes. At pyrimidine-ending (T/C) 2-fold synonymous sites (phenylalanine shown here) the only tRNA available has G opposite the 3^rd^ position of the codon, which binds NNC codons via Watson-Crick binding but NNU codons via G:U wobble, explaining the reduction in NNU codons in high expression genes. At purine-ending (A/G) 2-fold synonymous sites (glutamine shown here) both codons have cognate tRNAs but the NNG codon can also interact with the tRNA that has U opposite the 3^rd^ position of the codon (U:G wobble), explaining the reduction in NNG codons in high expression genes. (**C**) Competing forces at lysine codons in highly expressed genes. The AAA codon should be favoured because it can only bind its cognate tRNA (avoiding wobble interactions), while the AAG codon can bind a near-cognate tRNA via wobble interaction. However if the next codon begins with an adenine nucleotide, the AAA codon risks frameshifting.

According to the genetic code, the effect of neighbouring nucleotide context at 2-fold synonymous sites (third codon position) can be explored via the ratio of thymines:cytosines (T3:C3) (for the six pyrimidine-ending amino acid codon pairs) and adenines:guanines (A3:G3) (for the three purine-ending amino acid codon pairs) (Fig. 1A).

**Figure 2:**
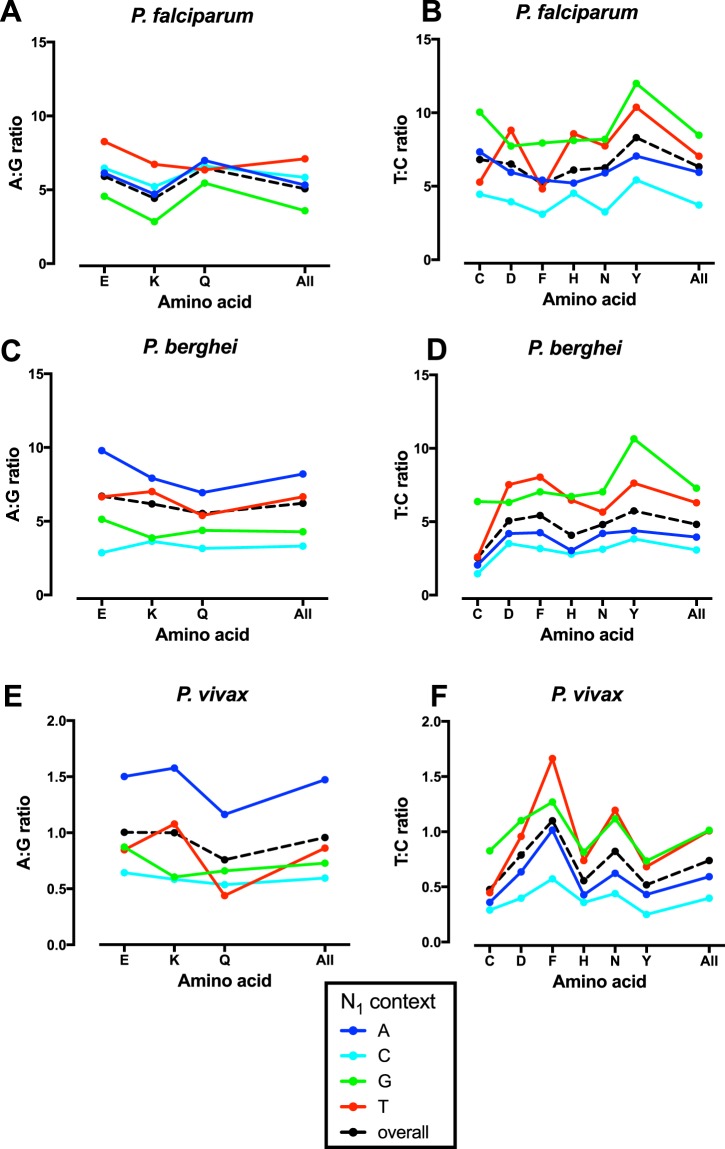
Effect of N_1_ context at 2-fold synonymous sites. A3:G3 and T3:C3 ratios at 2-fold synonymous sites are shown for *P*. *falciparum* (**A**,**B**), *P*. *berghei* (**C**,**D**) and *P*. *vivax* (**E**,**F**).

A clear association is present between N_1_ context and T3:C3 ratio in all three studied species (Fig. 2, Supplementary Table 1). Consistent with previous evidence of lower than expected CpG sites^29,32^, T3:C3 ratio is highest (and cytosines hence most rare) with N_1_G. For example, in *P*. *falciparum*, the T3:C3 ratio is 8.6 with N_1_G vs. 5.9 with N_1_H (H = ‘not G’) while in *P*. *berghei* T3:C3 is 7.3 with N_1_G vs. 4.4 with N_1_H. Incidentally the relatively low T3:C3 for cysteine in *P*. *berghei* agrees with previous results^36^.

It is also clear that T3:C3 is lowest (i.e. cytosines more common) when followed by another cytosine (N_1_C). This effect is consistent across all amino acids and all species (Fig. 2, Supplementary Table 1). In *P*. *falciparum*, T3:C3 ratio is 3.8 for N_1_C vs. 6.8 for N_1_D (D = ‘not C’). This suggests that there is protection of the positive strand C3 by the neighbouring cytosine nucleotide at N_1_. T3:C3 is also relatively low with N_1_A, perhaps because TA is suppressed.

N_1_ context is also associated with an altered A3:G3 ratio (Fig. 2, Supplementary Table 1**)**, although the effects are less consistent than with the T3:C3 ratio. In *P*. *falciparum* N_1_G is associated with the lowest A3:G3 ratio for all three amino acids (N_1_G = 3.6 vs. 5.7 with N_1_H overall), a potential explanation being protection of the cytosine at position 3 on the negative strand by its neighbouring 5′ cytosine. For *P*. *berghei* and *P*. *vivax* N_1_G is also associated with a low A3:G3 ratio (Supplementary Table 1), although A3:G3 is even lower (overall) with N_1_C, perhaps because AC is suppressed.

#### 4-fold sites (5 amino acids)

4-fold synonymous sites were also examined to see if the associations seen at 2-fold sites are present at 4-fold sites. In the absence of simple ratios we assessed associations between N_1_ context and the percentage of cytosines and guanines in the third position (C3% and G3%)(Supplementary Fig. 1, Supplementary Table 2).

As seen at 2-fold sites, cytosines are more rare with N_1_G for all species (6.2% vs. 9.2% with N_1_H for *P*. *falciparum*), consistent with CpG dinucleotides being reduced. Cytosines are also more common with N_1_C (C3% 10.2% vs. 8.3% for N_1_D) again suggesting protection of C3 on the positive strand by the 3′ N_1_C.

G3% is relatively less influenced by context at 4-fold sites. As at 2-fold sites, G3 is more common with N_1_G in *P*. *falciparum* (G3% 10.6% vs. 8.8% for N_1_H), consistent with protection of C3 on the negative strand by the neighbouring (5′) cytosine on the negative strand of the next codon. Smaller magnitude but highly significant effects in the same direction are also seen in *P*. *berghei* and *P*. *vivax*.

#### Within codon effects (4-fold sites, 5 amino acids)

The influence of N_1_ context within codons can be assessed by examining whether within 4-fold synonymous sites the presence of a cytosine at position 2 (C2; alanine, proline, threonine) is associated with nucleotide usage at the third position compared to codons without C2 (termed D2; glycine and valine). Two associations observed in the cross-codon data are also visible within codons of *P*. *falciparum* (Supplementary Fig. 2, Supplementary Table 3). C2 is associated with a low G3% (7.2% vs. 11.5% with D2), consistent with vulnerability of negative strand cytosines in the third position. C2 is also associated with a high C3% (11.0% with C2 vs. 5.6% with D2), consistent with protection of C3 on the positive strand by the 5′ C2 cytosine.

There are similar findings with *P*. *berghei* (G3% 6.9% with C2 vs. 14.0% with D2; C3% 10.7% with C2 vs. 7.8% with D2) and *P*. *vivax* (G3% 28.4% with C2 vs. 38.5% with D2; C3% 34.8% with C2 vs. 23.6% with D2) (Supplementary Fig. 2C–F).

### Expression-related codon bias

**Figure 3:**
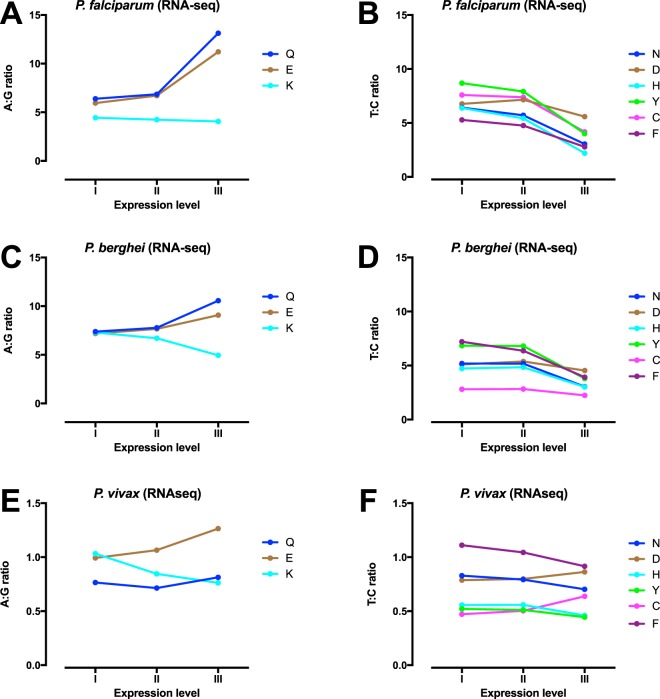
Effect of asexual gene expression at 2-fold synonymous sites. A3:G3 and T3:C3 ratios at 2-fold synonymous sites are shown for each of three expression levels for *P*. *falciparum* (**A**,**B**), *P*. *berghei* (**C**,**D**) and *P*. *vivax* (**E**,**F**). Results are presented for each individual amino acid.

We next examined the relationship between expression and codon bias in the three malaria parasite species, focussing for clarity on 2-fold synonymous sites where nucleotide bias can be expressed as a simple ratio. Based on previous work^28^, level of expression was grouped into three strata with the top stratum containing the top 3% of genes in terms of peak asexual phase expession. Intense expression in *P*. *falciparum* is generally associated with higher A3:G3 ratios (5.1 vs. 6.3 in the lowest and highest bands respectively), but lower T:C ratios (6.6 vs. 3.5) (Fig. 3A,B, Supplementary Tables 4 and 5). The fall in T3:C3 ratio with increasing expression is clear across all six relevant amino acids in *P*. *falciparum*, but the increase in A3:G3 ratio at higher levels of expression is not consistent, with the ratio approximately doubling for glutamatate and glutamine (producing a codon bias of more than 10-fold in the highest expression stratum) but falling significantly within lysine codons (4.4 to 4.1). There are similar findings using the *P*. *falciparum* microarray expression dataset (Supplementary Tables 4 and 5).

In *P*. *berghei*, there is a broadly similar picture, with consistent falls in T3:C3 ratio across all amino acids and similarly mixed findings at A3:G3 codons, with the fall in A3:G3 at lysine codons even more pronounced (Fig. 3C,D, Supplementary Tables 4 and 5). The preponderance of lysines compared to the other two amino acids means that in *P*. *berghei* the overall A3:G3 ratio at 2-fold A/G sites falls by around 12% in highly expressed genes.

For *P*. *vivax*, T3:C3 ratio only falls for some amino acids and the overall effect is of borderline significance. High level expression is associated with more significant changes in A3:G3 ratio, which rises in glutamate and glutamine codons but clearly falls at lysine codons.

In order to produce overall measures of selection for each organism, we calculated the strength of selection co-efficient *S* for the nine 2-fold synonymous codons, according to the approach of dos Reis and Wernisch^35^, comparing the proportion of the codon selected in the top expression band (III) with its proportion in the lowest expression band (I). *S* is the natural log of the odds ratio of the relative codon frequencies in highly expressed compared to reference genes. For *P*. *falciparum* values between 0.09 and 1.07 are obtained for the nine codons (Table 2), with broad correlation (Spearman correlation across nine amino acids = 0.67, p = 0.059) between our results and those previously reported for *P*. *falciparum*^35^ based on assumed expression levels of *P*. *falciparum* genes inferred by means of orthologous relationships (*S*. *cerevisiae* vs. *P*. *falciparum*).

**Table 2:** Calculations of the strength of codon selection coefficient *S* for the nine 2-fold synonymous codons for *P*. *falciparum* and *P*. *vivax*, along with analogous values obtained by dos Reis & Wernisch^35^.

Amino acid	Selected codon in *P*. *falciparum*	*P. falciparum*		*P. berghei*	*P. vivax*
		This study (RNA-seq data)	Dos Reis & Wernisch (inferred by orthology)	This study (RNA-seq data)	This study (RNA-seq data)
E	GAA	0.635	0.600	0.233	0.242
K	AAG	0.090	0.380	0.388	0.304
Q	CAA	0.720	0.420	0.358	0.061
C	TGC	0.598	1.100	0.225	0.301 (TGT)
D	GAC	0.192	0.290	0.118	0.092 (GAT)
F	TTC	0.641	0.530	0.610	0.194
H	CAC	1.067	1.030	0.450	0.192
N	AAC	0.744	1.350	0.534	0.165
Y	TAC	0.774	1.230	0.587	0.158
Averaged *S*		0.537		0.422	0.199

For two amino acids in *P*. *vivax* the direction of effect is opposite to that in *P*. *falciparum*.

For *P*. *berghei* the selection coefficients apply consistently in the same direction, with lower *S* values for most amino acids except lysine (see above). In *P*. *vivax*, *S* values are considerably smaller, varying from 0.092 to 0.304. Averaging across all amino acids (i.e. allowing for their relative frequencies in the entire coding sequence) we obtained averaged *S* values of 0.537 for *P*. *falciparum*, 0.422 for *P*. *berghei* and 0.199 for *P*. *vivax*.

### Stop codons and expression

The first observation is that underlying AT-bias clearly influences stop codon usage with TAA codons making up around two thirds of *P*. *falciparum* and *P*. *berghei* stop codons but only one fifth of *P*. *vivax*, consistent with the overall AT-richness in the three species.

**Figure 4:**
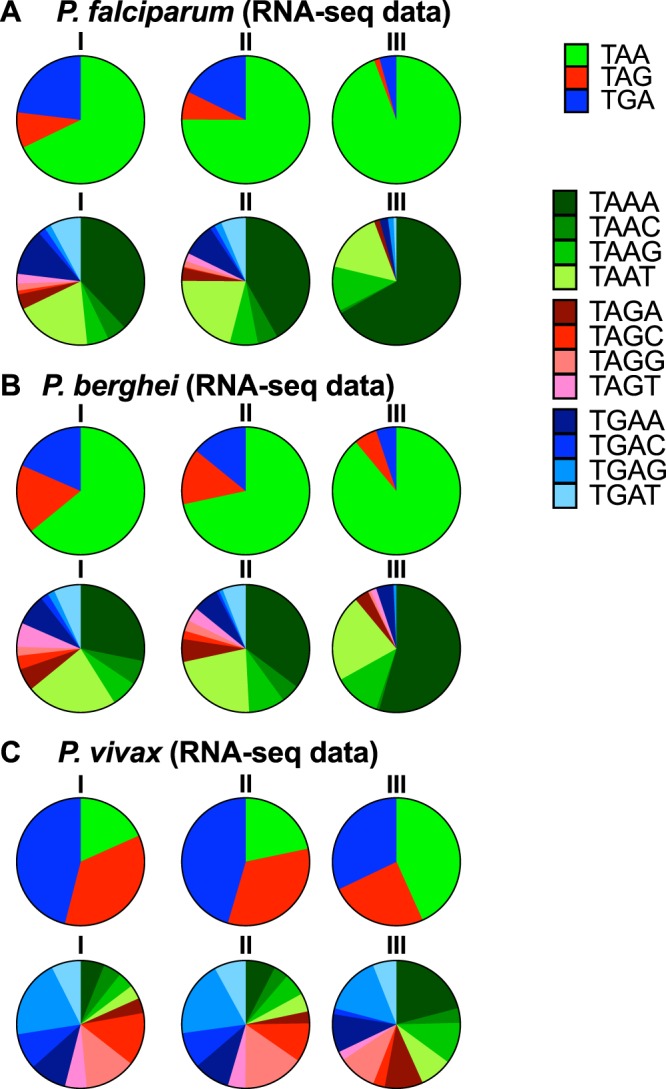
Effect of asexual gene expression and stop codon / tetranucleotide usage. Results are shown for each of three expression levels for *P*. *falciparum* (**A**), *P*. *berghei* (**B**) and *P*. *vivax* (**C**).

In *P*. *falciparum* the proportion of TAA stop codons increases from 67% to 94% in the top expression band while TAG and TGA codons fall significantly (Fig. 4, Supplementary Table 6). Tetranucleotides (the ‘traditional’ stop codon plus the following nucleotide) are also considered to be important in terms of efficiency of translational termination^37^. The TAAA tetranucleotide rises as a proportion of TAAN tetranucleotides (56% in the bottom expression band to 71% in the top) while TAAC and TAAT fall significantly and TAAG tetranucleotides show no significant change in proportion. Virtually identical associations were found when using ribosome profiling^24^ to quantify expression during the asexual lifecyle (Supplementary Table 6).

Highly analogous changes are seen with *P*. *berghei*, with TAA stop codons rising from 64% to 89% across expression bands; again, the TAAA tetranucleotide rises (as a proportion of TAA), from 44% to 61% while TAAC and TAAT undergo significant falls.

Changes in stop codon patterns of a similar direction, but smaller magnitude, are observed in *P*. *vivax* with TAA rising from 18% to 43% of stop codons in genes with high expression while TAG falls (Fig. 4, Supplementary Table 6). Within TAA tetranucleotides, there are again significant changes in TAAA proportion (rising with increased expression) and TAAC (falling) while TAAG and TAAT tetranucleotides are not significantly different.

### Combining intrinsic (AT-richness), contextual and expression-related biases

**Figure 5:**
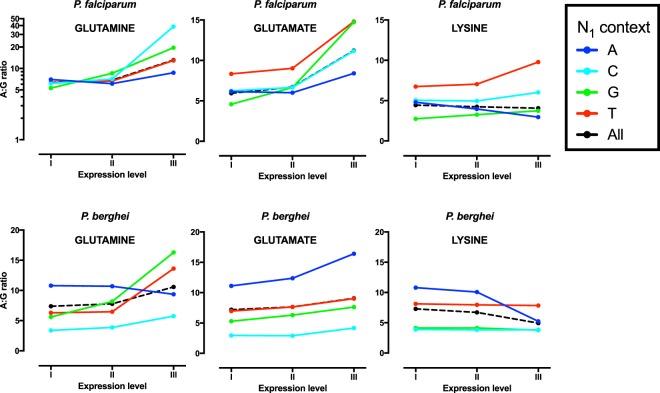
Contextual (N_1_) and expression-related effects on A3:G3 for 2-fold synonymous sites in *P*. *falciparum* and *P*. *berghei*. For glutamine in *P*. *falciparum* the A:G ratio is shown on a log-scale.

**Figure 6:**
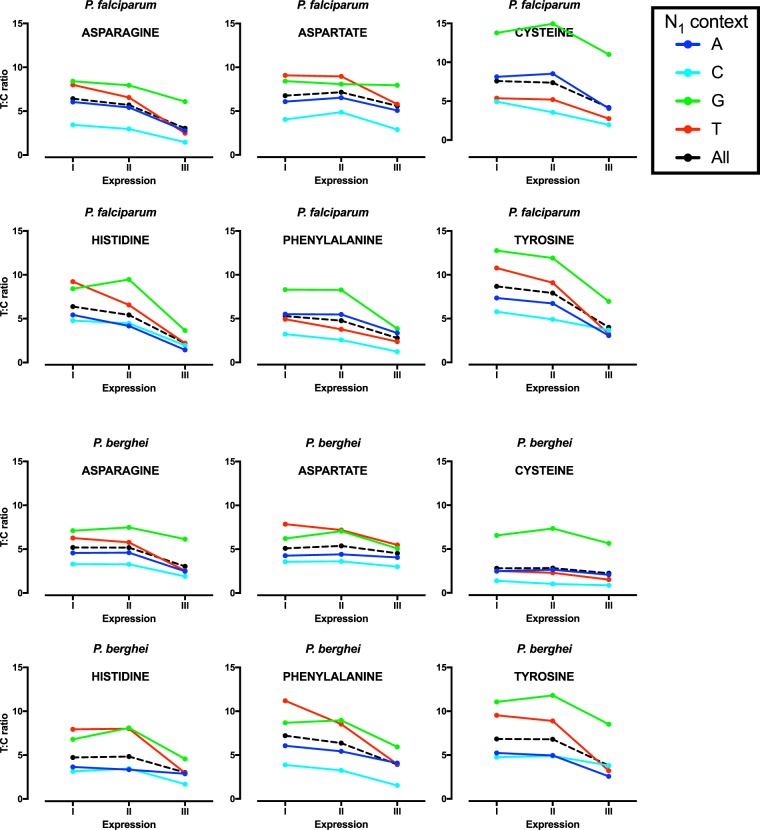
Contextual (N_1_) and expression-related effects on T3:C3 for 2-fold synonymous sites in *P*. *falciparum* and *P*. *berghei*.

Given the documented influence of overall AT-richness, local context and expression on codon bias in *P*. *falciparum*, we finally studied how these factors act in combination, in order to observe how overall AT-bias becomes more or less intense according to the prevailing set of conditions. For clarity of interpretation we again focused on changes in codon ratios at 2-fold synonymous sites (Figs 5 and 6).

The combination of underlying AT-richness and a high level of expression in *P*. *falciparum* are predicted to produce very high A3:G3 ratios; in glutamine and glutamate codons in highly expressed genes A3:G3 ratio is greater than 10, and for glutamine followed by N_1_C it increases to 38.8. As noted above, lysine codons behave differently; importantly the distinctive overall fall in A3:G3 ratio associated with high expression at lysine codons (see above) appears essentially attributable to codons where the following codon starts with an adenine (N_1_A, which is present after 52% of all *P*. *falciparum* lysine codons). In this context A3:G3 falls from 4.8 in the lowest expression stratum to 3.0 in the highest. In other contexts (N_1_B) A3:G3 in lysine codons rises with expression, in line with glutamine and glutamate. In *P*. *berghei* also, N_1_ context clearly interacts with expression-related effects, with the N_1_A context again explaining much of the distinctive fall in A3:G3 ratio in lysines of highly expressed genes (with N_1_A, A3:G3 falls from 10.8 in the lowest expression stratum to 5.2 in the highest).

The combined effects of N_1_ context and the influence of expression on T3:C3 ratio in *P*. *falciparum* are also generally predictable from the individual effects (Fig. 6). The lowering of T3:C3 ratio in intensely expressed genes is countered in the N_1_G context (so that T3:C3 remains above 3 in all cases). However with N_1_C and intense expression (factors independently associated with preservation of C3), the two forces act in the same direction to lower T3:C3 ratio, producing an overall T3:C3 ratio across all codons of only 1.9. This is most extreme in phenylalanine codons followed by N_1_C, which in highly expressed genes have a T3:C3 ratio of only 1.22. Similar patterns are observed with *P*. *berghei*, with the T:C ratio in an N_1_C context reduced to 2.0 (across all six amino acids) and the AT-bias at cysteines followed by N_1_C disappearing entirely in highly expressed genes (T3:C3 = 0.87).

## Discussion

Genomewide association studies focus on non-synonymous changes, with synonymous mutations typically not considered as likely causes of phenotypic change^38^. In previous work we showed that many non-synonymous polymorphisms in the non-conserved sections of *P*. *falciparum* coding sequence are likely to be non-adaptive in nature^39^, emphasising the importance of neutral evolution in *P*. *falciparum* and suggesting how taking this into account might be of benefit in assessing drug-resistance mutations in population genetic studies of *P*. *falciparum*^40^. In contrast, this work, and other key studies which preceded it^27,28,41^, indicate that synonymous mutations in highly expressed genes (including their stop sequences) are not simply neutral.

This study builds on previous observations on codon bias in malaria parasites, revealing how codon bias is influenced by several complex ‘layers’ of mutational and selective forces that differ between malaria species. The most obvious (and ‘deepest’) layer of bias is the overall AT-richness of the genome, with extreme AT-richness found in both the Laverania group (exemplified by *P*. *falciparum*) and rodent parasites (e.g. *P*. *berghei*). In *P*. *falciparum* this property is maintained by a mutational force, with both population genetic studies^42^ and studies of mutation in continuous cultures^43^ indicating that the genome is broadly in equilibrium, although this bias might originally have been selected.

A second clear ‘layer’ of influence on codon bias is local DNA context. Irrespective of the underlying AT-bias of each genome, cytosines are the most vulnerable nucleotide^44^ and the probability of mutation (presumably via deamination) is context-dependent. There is a high T3:C3 ratio at synonymous positions followed by a 3’ guanine nucleotide, consistent with CpG dinucleotide suppression, a finding previously reported in focused^29,31^ and genome-wide studies^32^. In organisms with DNA methylation, CpG suppression is frequently attributed to enhanced spontaneous deamination of methylated cytosines (5-mC) to thymine, which results in a T:G mismatch that can then be repaired, or replicated, to give a C:G to T:A substitution. However there is conflicting evidence as to whether DNA methylation occurs in malaria parasites^30,45,46^ and other Apicomplexans^47^. If the CpG suppression is not due to methylation, it may simply represent a methylation-independent mutational hotspot. CpG suppression clearly occurs in yeast in the absence of DNA methylation^48^.

Mutational bias in the absence of methylation would also explain other distinct contextual effects that we observe. In contrast to the vulnerability of cytosine preceding guanines, cytosines appear to be protected when another cytosine is neighbouring, consistent with the previous finding that in coding regions of a number of *P*. *falciparum* genes, CC dinucleotides are more common than expected^29^. The effect is particularly strong when a cytosine is found on the 3’ side. In all species and for all amino acids, the T3:C3 ratio at 2-fold synonymous codons is reduced with the presence of an N_1_ cytosine; there is additional supportive evidence from 4-fold codons. We also note that a 5′ cytosine protects cytosines, with higher levels of G3 with N_1_G, consistent with protection of C3 on the negative strand by the neighbouring C on the negative strand of the next codon (5′). This is strengthened further by within-codon data, showing that the 4-fold amino acids with C2 (alanine, proline and threonine) have a signficantly higher proportion of C3 than others (glycine and valine). Additional effects, such as T3:C3 being relatively low with N_1_A, and A3:G3 being lowest with N_1_G in *P*. *berghei* and *P*. *vivax*, are consistent with previous work on dinucleotides^29^ and indicate that a variety of local contextual forces influence synonymous positions. These contextual findings are important when interpreting changes in codon bias in genes with a high level of expression in the asexual cycle (see below).

Our study of the influence of expression on codon bias^27,28,35^ extends previous work by including more recent RNA-seq data pertaining to three malaria species. As previously reported for *P*. *falciparum*, high level expression in *P*. *falciparum* and *P*. *berghei* is associated with a broad increase in A3:G3 ratio and reduction in T3:C3 ratio at 2-fold synonymous sites^27,28^. Assuming that selection drives these effects, what are the likely mechanisms? Musto *et al*., who first described distinctive patterns of codon usage according to level of gene expression^27^, suggested that the selective force could be optimisation of translation according to available tRNAs, since for pyrimidine (C or T-ending codons) the only existing isoacceptor tRNA binds perfectly with the significantly incremented triplet^28^ (Fig. 1B). Recent work by Chan *et al*. during the conduct of our study^41^ explores this further, in particular at asparagine codons where AAT codons can only be decoded via a near-cognate tRNA predicted to bind only via a ‘wobble’ interaction^49^. Reading of codons by wobble binding in metazoans is associated with slower translation because of ribosomal stalling^11^. By studying asparigine homorepeats, Chan *et al*. were able to show directly that wobble pairings also reduce translation efficiency in *Plasmodium falciparum*^41^. Hence the fall in T3:C3 ratio in high expression genes (also reported by Chan *et al*. to be greater in *P*. *falciparum* than *P*. *vivax*) is readily explained by selection against such codons.

For purine (A- or G-ending) codons, malaria parasites have tRNA isoacceptors cognate for both possible codons. The ‘wobble’ hypothesis might again be relevant, since any NNG codons can not only bind their cognate C-starting tRNA anticodon, but also the near-cognate U-starting tRNA anticodon (see Fig. 1B), via wobble interaction. Increased use of NNA codons that bind only their cognate tRNA would prevent such pairing (with its attendant reduction in translational efficiency), providing a selective hypothesis for the higher A3:G3 ratio. Avoidance of wobble interactions might also contribute to the higher A3:G3 ratios in the highly expressed genes of certain other organisms; for example in *S*. *cerevisiae*, for Gln there are 9 UUG tRNAs (cognate to CAA codons) and only one tRNA for CUG (cognate to CAG codons)^35^; selection of CAA, which can only bind the UUG tRNAs, would reduce the number of wobble interactions.

In *P*. *vivax* the same overall changes take place in high expression genes, although they are of lesser magnitude and less consistent across the various amino acids in each group. This fits with data from Chan *et al*. which report minimal reduction in use of wobble codons in highly expressed *P*. *vivax* genes^41^.

An interesting exception to the broad patterns of codon bias associated with high gene expression is the clear fall in A3:G3 ratio in lysine codons; this is evident for all three malaria species studied, being most clearly visible in *P*. *berghei*. Chan *et al*. also found that lysine codons have distinctive properties (lower use of A-ending codons) in ribosomal proteins, considered as reference genes for describing optimal codon usage. How can this pattern be explained? Local DNA context appears to be critical, since in both *P*. *falciparum* and *P*. *berghei* the presence of an adenine at the start of the next codon (N_1_A) is associated with substantially lower A3:G3 ratio in high expression genes, while the other three N_1_ possibilities produce more typical increases in A3:G3 ratio. A logical explanation for this is that in highly expressed genes, where there is selection for the AAA codon (binding the single UUU isoacceptor hence avoiding wobble binding), there is also competing selection against the AAAA tetranucleotide, since after binding of the UUU isoacceptor tRNA there can be + 1 frameshifting (Fig. 1C). Around half of lysine codons are followed by an A at the start of the next codon so this is a significant force. Frameshifting at lysine codons has been described in bacteria^8^, and frequent translational errors of this form would clearly produce devastating consequences in highly expressed genes^50^, with waste of energy to generate and then degrade high levels of non-functional peptide chains; misfolded proteins also risk cell toxicity^51^.

We also studied stop codon usage, and how this changes in high expression genes, with the idea that this might also provide an insight into generalised forces acting on DNA, as well as the distinctive biology of translational termination^5^. Forces acting upstream or downstream of translation should apply equally to stop and sense codons, whereas if selection on codon bias is principally associated with tRNA frequencies or interactions, the stop codons may behave differently in highly expressed genes. The first observation was that TAA codons make up around two thirds of *P*. *falciparum* and *P*. *berghei* stop codons but only one fifth of *P*. *vivax*, consistent with the overall AT-richness in the three species. In all three species of malaria parasites, highly expressed genes show increasing use of the TAA codon, with the bias in *P*. *falciparum* and *P*. *berghei* particularly striking (the proportion of TAA codons rising to 94 and 89% respectively). This is of interest as across a wide range of species such relationships between expression and stop codon usage have been hard to discern^52^, and suggests that the (presumably) selective forces are relatively strong in these malaria species. TAA is also favoured in highly expressed genes in humans^53^. The simplest mechanistic explanation for increasing TAA relates to efficiency^54^, as TAA is the ‘universal’ stop codon and binds to either of the relevant release factors, a phenomenon well documented in bacteria^55^.

Translational termination is further optimised by the flanking base at the downstream position (+1) so that the ‘stop tetranucleotide’ can be considered to signal the termination of protein synthesis in eukaryotes^37^. In highly expressed genes of all three malaria species, TAAA tetranucleotides occupy a significantly higher proportion within the overall set of TAA trinucleotide stop codons, while TAAC (and TAAT in *P*. *falciparum* and *P*. *berghei*) become significantly less common; TAAG is not significantly changed. These findings match those obtained in mammalian systems where the order for termination efficiency of the base at +1 position was found to be A ≈ G ≫ C ≈ U (independently of the stop codon), UAAA being the most efficient four-base combination^56^.

These expression-related effects are likely to promote fitness by improving efficiency of translation and hence producing more competitive parasites. Their strength differs according to species; we find that selection parameter *S* (averaged across amino acids) is largest in *P*. *falciparum*, slightly smaller in *P*. *berghei* and substantially smaller again in *P*. *vivax*, with values generally lower than for other typical eukaryotic genomes^35^. We note that this ranking matches the relative overall AT-richness of the respective genomes. This correlation, which could potentially be explored across a wider range of malaria species, suggests that AT-richness itself might also be a fitness adaptation promoting replication ‘efficiency’; for example the genomes of bacteria that rely on their host for survival tend to be AT-rich given the increased energy cost in GTP and CTP synthesis^57^. Hence overall AT-richness, codons that avoid tRNA wobble interactions and frameshifts, and specific stop sequences may all provide small competitive advantages in terms of speed and efficiency of asexual replication.

Why do these processes operate at different levels in the different malaria species? The answer might relate to lifecycle biology, which has distinct characteristics according to parasite species. One prominent example is the relatively early gametocytogenesis of human *P*. *vivax* infection, in which transcriptional control promotes early conversion to the sexual stage; this might therefore reduce selection for maximal asexual cycle efficiency^58^. Alternatively, the effective population size of the organism over evolutionary time might be responsible for the differences in synonymous codon usage. The probability that a mutation will change in frequency depends on the product of the effective population size and the true selection coefficient (*s*)^59^ (the parameter *S* which we describe above is actually a ‘confounded’ selection co-efficient that is influenced by effective population size). There are several examples of codon usage bias being determined by effective population size in other eukaryotes^60^, with shifts in codon bias tending to occur when organisms have a large effective population size that facilitates the selection of mild-effect polymorphisms^35^. By contrast, a prolonged population bottleneck may result in reduced selection and low levels of codon bias even in highly expressed genes. There have been substantial advances in our understanding of the evolutionary histories of malaria parasites^61^ in recent years, but a description of how effective population size has varied in each species and its ancestors remains some way off. Interestingly, a recent study based on MalariaGEN Community Project data suggests a substantially higher preference for AT nucleotides compared to GC nucleotides at synonymous single nucleotide polymorphism sites in *P*. *vivax*^62^, a process that in principle should produce a more AT-rich genome over time. This is consistent with the finding that *P*. *vivax* has recently undergone a population expansion relative to *P*. *falciparum*^58,63^.

### Limitations

Our study was of descriptive design and hence did not meet all criteria allowing distinction between association and causality, particularly relevant if selective forces are being invoked^64^. It is therefore appropriate to consider whether there might be mutational explanations for phenomena where we propose selective hypotheses. For example, bacterial expression can influence codon usage because the DNA of highly expressed genes spends a relatively greater proportion of its time in single-stranded form, making it more prone to mutational forces^65^. Strand bias in cytosine deamination has been postulated to play a major role in genome evolution^66^ with cytosines less common in the non-transcribed strand of highly expressed genes; however this is the opposite of the pattern we observe in *P*. *falciparum*. The similarity between the patterns of codon bias we found in highly expressed genes, and analogous studies in other higher eukaryotes^35^, also supports selective explanations.

In terms of the influence of local DNA context on codon bias, we assume the opposite i.e. that context introduces mutational ‘hotspots’ (CpG) or ‘coldspots’ (CpC). One factor supporting this assumption is that effects appear consistent across multiple amino acids, and species, and follow simple rules at the DNA level. Again, direct evidence that mutational force mediates these contextual effects remains lacking. There appears to be no statistically significant enrichment of CpG or TpC dinucleotides at de novo C → T transitions in cultured *P*. *falciparum* lines^43^, although the power of that study was probably not sufficient to discern an additional contextual effect beyond the underlying AT-bias. Large scale population genetic data might shed more light on this area^67^.

Our study necessarily focused on certain categories of variation in DNA context or expression. We looked at a series of neighbouring base contextual effects, but the constraints of the genetic code meant that certain contexts were not examined comprehensively, including preceding (−1) bases, and more distant bases. For studies of codon bias in highly expressed genes we focused on 2-fold codons as interpretation is relatively simpler than in 4-fold sites where complexity of competing forces is likely to make interpretation challenging. Levels of protein expression may also be influenced by other adaptive changes, for example in RNA stability and tRNA modification^68^, factors not explored in this study.

Our primary analyses focused upon the transcriptome as assessed by RNA-seq methodology, given the availability of comparable datasets for all three studied species. In the case of *P*. *falciparum* we were able also to look at the association between codon bias and level of mRNA translation using ribosome profiling data^24^, comparing the results to data from RNA-seq.^20^; for convenience we focused on stop codons. Given the tight coupling between transcription and translation, particularly for the core genome^24^, it was no surprise to see virtually identical results across RNA-seq and ribosome profiling data, confirming that TAA stop codon (and the TAAA tetranucleotide) is enriched in genes that are highly transcribed and translated. Our method to rank genes by level of expression was relatively simple and based on previous work, taking the maximum level expression at any point in the asexual cycle as a particular gene’s value for ranking purposes^28^. In their recently published work, Chan *et al*. examined the top 5% expressed genes during the progression of the asexual cycle in a highly analogous approach to ours^41^.

## Conclusions

The various forms of codon bias in malaria parasites result from a complex set of forces that produce a variegated bias within and between genes. Local DNA context exerts clear effects, with CpG dinucleotides associated with cytosine depletion and CpC dinucleotides relative cytosine protection. Genes that are expressed intensely during asexual stages are affected by additional forces that are consistent with promotion of translational efficiency through avoidance of wobble interactions during tRNA binding, reduction of frameshifting and optimisation of termination. These expression-related biases are more substantial in the AT-rich genomes of *P*. *falciparum* and *P*. *berghei* than in the less AT-rich genome of *P*. *vivax*, suggesting that underlying properties of the different species, such as the competitive advantage offered by rapid replication or overall population size, influence both AT-richness and codon bias.

## Electronic supplementary material

Supplementary Information
