# Supplementary material for: Forces acting on codon bias in malaria parasites
Source: Sci Rep. 2018 Oct 29;8:15984. doi: 10.1038/s41598-018-34404-9 (PMC6206010; doi:10.1038/s41598-018-34404-9)
Supplement: Supplementary file 1 — Supplementary Information [file 41598_2018_34404_MOESM1_ESM.doc]

**Forces acting on codon bias in malaria parasites**

Sinha I^1,2^ , Woodrow CJ^1,2^

^1^Mahidol-Oxford Tropical Medicine Research Unit (MORU), Mahidol University, Bangkok, Thailand.

^2^Centre for Tropical Medicine and Global Health, University of Oxford, UK

Supplementary Data

| Species | T3:C3 with N_1_G | T3:C3 with N_1_H | % change in codon bias* | T3:C3 with N_1_C | T3:C3 with N_1_D | % change in codon bias* | A3:G3 with N_1_G | A3:G3 with N_1_H | % change in codon bias* |
| --- | --- | --- | --- | --- | --- | --- | --- | --- | --- |
| *P. falciparum* | 8.59 | 5.94 | 30.9% | 3.77 | 6.81 | -80.5% | 3.59 | 5.74 | -59.9% |
| *P. berghei* | 7.28 | 4.41 | 39.4% | 3.07 | 5.07 | -65.1% | 4.29 | 6.83 | -59.2% |
| *P. vivax* | 1.01 | 0.64 | 36.6% | 0.40 | 0.83 | -107.5% | 0.73 | 1.08 | -47.9% |

**Supplementary Table 1** – Cross codon contextual effects at all 2-fold sites according to the presence of cytosine or guanine at the first position of the following codon (N_1_C or N_1_G). In each case the effect is expressed in terms of the % change in codon bias associated with N_1_C or N_1_G (see Panel 1A). *p<0.0001 for all comparisons by Chi-squared test.

| Species | C3% with N_1_G | C3% with N_1_H | % change in C* | C3% with N_1_C | C3% with N_1_D | % change in C* | G3% with N_1_G | G3% with N_1_H | % change in C* |
| --- | --- | --- | --- | --- | --- | --- | --- | --- | --- |
| *P. falciparum* | 6.22 | 9.24 | -32.6% | 10.18 | 8.33 | 22.2% | 10.59 | 8.75 | 20.9% |
| *P. berghei* | 7.68 | 9.83 | -21.8% | 14.76 | 8.76 | 68.5% | 10.60 | 9.87 | 7.4% |
| *P. vivax* | 22.65 | 32.76 | -30.9% | 34.88 | 28.24 | 23.5% | 35.06 | 32.30 | 8.5% |

**Supplementary Table 2** – Cross codon contextual effects at all 4-fold sites according to the presence of cytosine or guanine at the first position of the following codon (N_1_C, N_1_G). *p<0.0001 for all comparisons on Chi-squared test.

| Species | G3% with C2 | G3% with D2 | % change in G* | C3% with C2 | C3% with D2 | % change in C* |
| --- | --- | --- | --- | --- | --- | --- |
| *P. falciparum* | 7.23 | 11.51 | -37.2% | 10.99 | 5.61 | 95.8% |
| *P. berghei* | 6.94 | 13.98 | -50.4% | 10.71 | 7.78 | 37.7% |
| *P. vivax* | 28.36 | 38.51 | -26.3% | 34.80 | 23.60 | 47.5% |

**Supplementary Table 3** – Within codon contextual effects on the content of the third nucleotide at 4-fold synonymous sites, stratified by presence vs. absence of cytosine at the second position (C2 vs. D2). *p<0.0001 for all comparisons on Chi-squared test.

| *P. falciparum* (RNA-seq) | I | III | Fold change | p |  | *P. falciparum*  (Microarray) | I | III | Fold change | p |
| --- | --- | --- | --- | --- | --- | --- | --- | --- | --- | --- |
| C | 7.59 | 4.17 | -45.0% | 1.44x10^-08^ |  | C | 7.07 | 5.35 | -24.3% | 1.71x10^-09^ |
| D | 6.77 | 5.59 | -17.4% | 3.15x10^-05^ |  | D | 6.75 | 5.90 | -12.6% | 5.62x10^-05^ |
| F | 5.28 | 2.78 | -47.3% | 1.69x10^-39^ |  | F | 5.26 | 3.43 | -34.8% | 2.84x10^-22^ |
| H | 6.38 | 2.19 | -65.6% | 2.05x10^-63^ |  | H | 6.26 | 2.88 | -54.1% | 2.19x10^-45^ |
| N | 6.42 | 3.05 | -52.5% | 2.65x10^-90^ |  | N | 6.37 | 4.71 | -26.0% | 2.12x10^-25^ |
| Y | 8.68 | 4.01 | -53.9% | 5.29x10^-41^ |  | Y | 8.44 | 5.29 | -37.3% | 2.62x10^-27^ |
| Overall | 6.65 | 3.46 | -47.9% | 1.34x10^-200^ |  | Overall | 6.57 | 4.59 | -30.1% | 3.51X10^-93^ |
|  |  |  |  |  |  |  |  |  |  |  |
| *P. berghei* (RNA-seq) | I | III | Fold change | p |  |  |  |  |  |  |
| C | 2.81 | 2.24 | -20.2% | 0.054 |  |  |  |  |  |  |
| D | 5.10 | 4.53 | -11.1% | 0.004 |  |  |  |  |  |  |
| F | 7.21 | 3.92 | -45.6% | 1.19x10-^21^ |  |  |  |  |  |  |
| H | 4.73 | 3.01 | -36.2% | 1.98x10-^05^ |  |  |  |  |  |  |
| N | 5.18 | 3.04 | -41.4% | 1.93x10-^24^ |  |  |  |  |  |  |
| Y | 6.83 | 3.80 | -44.4% | 1.76x10-^15^ |  |  |  |  |  |  |
| Overall | 5.40 | 3.49 | -35.3% | 2.32x10-^52^ |  |  |  |  |  |  |
|  |  |  |  |  |  |  |  |  |  |  |
| *P. vivax* (RNA-seq) | I | III | Fold change | p |  | *P. vivax*  (Microarray) | I | III | Fold change | p |
| C | 0.47 | 0.64 | 35.1% | 2.34x10^-04^ |  | C | 0.47 | 0.57 | 21.4% | 1.62x10^-04^ |
| D | 0.79 | 0.86 | 9.7% | 0.097 |  | D | 0.78 | 0.84 | 8.4% | 5.61x10^-12^ |
| F | 1.11 | 0.92 | -17.6% | 2.69x10^-06^ |  | F | 1.10 | 1.15 | 5.0% | 0.198 |
| H | 0.56 | 0.46 | -17.5% | 0.078 |  | H | 0.55 | 0.59 | 6.0% | 0.001 |
| N | 0.83 | 0.70 | -15.2% | 1.74x10^-06^ |  | N | 0.83 | 0.81 | -1.6% | 0.184 |
| Y | 0.52 | 0.44 | -14.7% | 0.031 |  | Y | 0.52 | 0.54 | 4.1% | 0.059 |
| Overall | 0.74 | 0.69 | -6.6% | 0.005 |  | Overall | 0.73 | 0.78 | 5.6% | 1.67x10^-12^ |

**Supplementary Table 4** – T3:C3 ratios according to level of expression for six amino acids, and two methods of measuring expression (for *P. falciparum* and *P. vivax*).

| *P. falciparum* (RNA-seq) | I | III | Fold change | p | *P. falciparum* (Microarray) | I | III | Fold change | p |
| --- | --- | --- | --- | --- | --- | --- | --- | --- | --- |
| E | 5.94 | 11.22 | 88.7% | 2.4x10^-31^ | E | 6.00 | 8.92 | 48.8% | 2.33x10^-21^ |
| K | 4.44 | 4.06 | -8.6% | 8.18x10^-05^ | K | 4.51 | 3.88 | -13.9% | 5.13x10^-18^ |
| Q | 6.39 | 13.13 | 105.4% | 1.19x10^-12^ | Q | 6.38 | 10.39 | 62.8% | 7.68x10^-12^ |
| Overall | 5.06 | 6.31 | 24.7% | 2.16E-14 | Overall | 5.12 | 5.73 | 12.0% | 3.65x10^-09^ |
|  |  |  |  |  |  |  |  |  |  |
| *P. berghei* (RNA-seq) | I | III | Fold change | p |  |  |  |  |  |
| E | 7.19 | 9.08 | 26.3% | 7.19x10^-05^ |  |  |  |  |  |
| K | 7.29 | 4.94 | -32.2% | 1.00x10^-22^ |  |  |  |  |  |
| Q | 7.38 | 10.57 | 43.2% | 0.0028 |  |  |  |  |  |
| Overall | 7.27 | 6.39 | -12.1% | 7.68x10^-04^ |  |  |  |  |  |
|  |  |  |  |  |  |  |  |  |  |
| *P. vivax* (RNA-seq) | I | III | Fold change | p | *P. vivax*  (Microarray) | I | III | Fold change | p |
| E | 0.99 | 1.26 | 27.4% | 8.82x10^-16^ | E | 0.99 | 1.13 | 14.3% | 2.47x10^-18^ |
| K | 1.03 | 0.76 | -26.2% | 1.85x10^-92^ | K | 1.02 | 0.92 | -10.3% | 1.18x10^-24^ |
| Q | 0.77 | 0.81 | 6.3% | 2.91x10^-04^ | Q | 0.76 | 0.81 | 6.8% | 1.09x10^-01^ |
| Overall | 0.97 | 0.91 | -6.1% | 2.33x10^-24^ | Overall | 0.99 | 1.13 | 14.3% | 2.47x10^-18^ |

**Supplementary Table 5** – A3:G3 ratios according to level of expression for three amino acids, two species and two methods of measuring expression.

|  |  |  | Expression level | | |  |
| --- | --- | --- | --- | --- | --- | --- |
|  |  |  | I | II | III | p value |
| *P. falciparum* stop codon **RNA-seq** | TAA |  | 2396 (67.8%) | 778 (75.1%) | 133 (94.3%) | 7.06 x 10^-14^ |
|  | TAG |  | 320 (9.1%) | 74 (7.1%) | 2 (1.4%) | 0.001 |
|  | TGA |  | 816 (23.1%) | 184 (17.8%) | 6 (4.3%) | 3.51 x 10^-09^ |
|  | *S* (TAA) |  |  |  | *2.06* |  |
|  |  |  |  |  |  |  |
|  | TAA Stop ‘Tetranucleotide’ | TAAA | 1339 (55.9%) | 433 (55.7%) | 94 (70.7%) | 0.003 |
|  |  | TAAC | 175 (7.3%) | 53 (6.8%) | 1 (0.8%) | 0.013 |
|  |  | TAAG | 194 (8.1%) | 74 (9.5%) | 16 (12.0%) | 0.17 |
|  |  | TAAT | 688 (28.7%) | 218 (28.0%) | 22 (16.5%) | 0.01 |
|  | *S* (TAAA) |  |  |  | *0.64* |  |
|  |  |  |  |  |  |  |
|  |  |  | I | II | III |  |
| *P. falciparum* stop codon **Ribosome profiling** | TAA |  | 1684 (67.1%) | 569 (77.2%) | 93 (93.0%) | 2.1 x 10^-12^ |
|  | TAG |  | 221 (8.8%) | 48 (6.5%) | 2 (2.0%) | 0.01 |
|  | TGA |  | 606 (24.1%) | 120 (16.3%) | 5 (5.0%) | 6.5 x 10^-09^ |
|  | *S* (TAA) |  |  |  | *1.88* |  |
|  |  |  |  |  |  |  |
|  | TAA Stop ‘Tetranucleotide’ | TAAA | 920 (54.6%) | 330 (58.0%) | 72 (77.4%) | 6 x 10^-05^ |
|  |  | TAAC | 136 (8.1%) | 30 (5.3%) | 0 (0%) | 0.002 |
|  |  | TAAG | 132 (7.8%) | 54 (9.5%) | 10 (10.8%) | 0.33 |
|  |  | TAAT | 496 (29.4%) | 155 (27.2%) | 11 (11.8%) | 0.001 |
|  | *S* (TAAA) |  |  |  | *1.05* |  |
|  |  |  |  |  |  |  |
|  |  |  | I | II | III |  |
| *P. berghei*  stop codon **RNA-seq** | TAA |  | 2198 (64.1%) | 679 (71.6%) | 121 (89.0%) | 8.74x10^-12^ |
|  | TAG |  | 599 (17.5%) | 135 (14.2%) | 8 (5.9%) | 2.04x10^-04^ |
|  | TGA |  | 633 (18.5%) | 134 (14.1%) | 7 (5.15%) | 6.24x10^-06^ |
|  | *S* (TAA) |  |  |  | *1.51* |  |
|  |  |  |  |  |  |  |
|  | TAA Stop ‘Tetranucleotide’ | TAAA | 966 (43.9%) | 334 (49.2%) | 74 (61.2%) | 1.46x10^-04^ |
|  |  | TAAC | 214 (9.7%) | 45 (6.6%) | 1 (0.8%) | 3.14x10^-04^ |
|  |  | TAAG | 230 (10.4%) | 87 (12.8%) | 16 (13.2%) | 0.176 |
|  |  | TAAT | 788 (35.9%) | 213 (31.4%) | 30 (24.8%) | 0.008 |
|  | *S* (TAAA) |  |  |  | *0.70* |  |
|  |  |  |  |  |  |  |
|  |  |  | I | II | III |  |
| *P. vivax*  stop codon **RNA-seq** | TAA |  | 618 (18.3%) | 215 (21.7%) | 58 (43.3%) | 2.38 x 10^-12^ |
|  | TAG |  | 1204 (35.7%) | 325 (32.8%) | 33 (24.6%) | 0.01 |
|  | TGA |  | 1553 (46.0%) | 450 (45.5%) | 43 (32.1%) | 0.01 |
|  | *S* (TAA) |  |  |  | *1.23* |  |
|  |  |  |  |  |  |  |
|  | TAA Stop ‘Tetranucleotide’ | TAAA | 205 (33.2%) | 75 (34.9%) | 28 (48.3%) | 0.07 |
|  |  | TAAC | 155 (25.1%) | 37 (17.2%) | 5 (8.6%) | 0.002 |
|  |  | TAAG | 137 (22.2%) | 57 (26.5%) | 14 (24.1%) | 0.427 |
|  |  | TAAT | 121 (19.6%) | 46 (21.4%) | 11 (19.0%) | 0.832 |
|  | *S* (TAAA) |  |  |  | *0.63* |  |

**Supplementary Table 6** Numbers and proportions of stop codons and TAA stop tetranucleotides according to level of expression in three malaria species (RNA-seq data as well as ribosome profiling data for *P. falciparum*). Strength of selection coefficients *S* are shown for the proportion of TAA stop codons and TAAA tetranucleotides (out of all TAA stop codons) comparing expression bands I and III.

**Supplementary Figure 1:** Effect of N_1_ context at 4-fold synonymous sites for five amino acids (alanine, glycine, proline, threonine and valine). C3% and G3% ratios are shown for *P. falciparum* (A, B), *P. berghei* (C, D) and *P. vivax* (E,F).

**Supplementary Figure 2**: C3% and G3% at 4-fold synonymous sites, according to the identity of the second nucleotide in the codon. Data are obtained for *P. falciparum* (A, B), *P. berghei* (C, D) and *P. vivax* (E,F).
